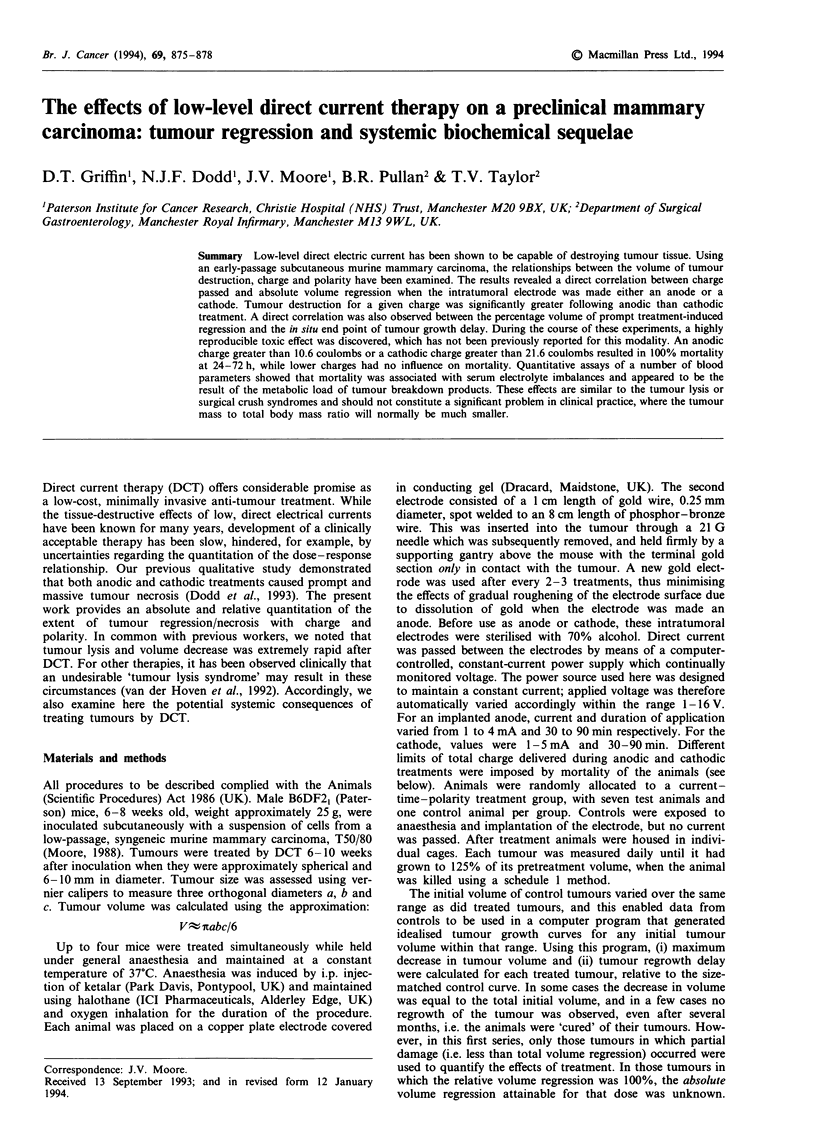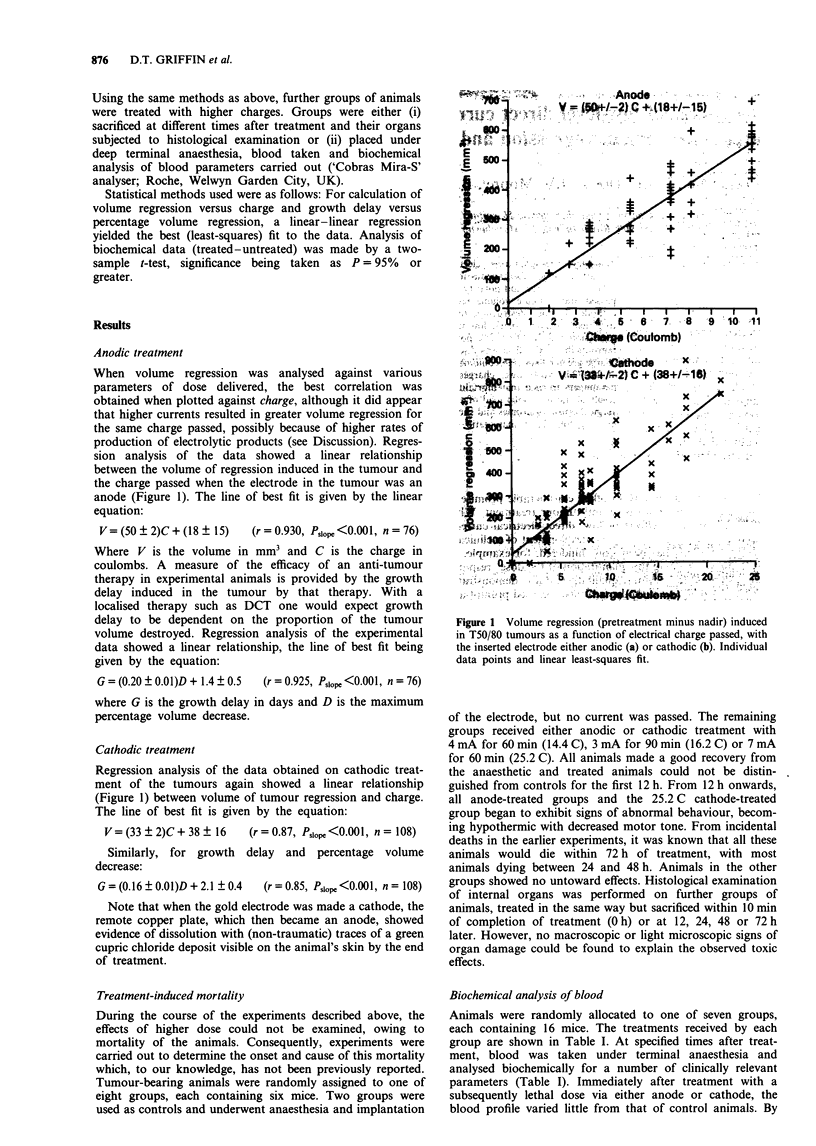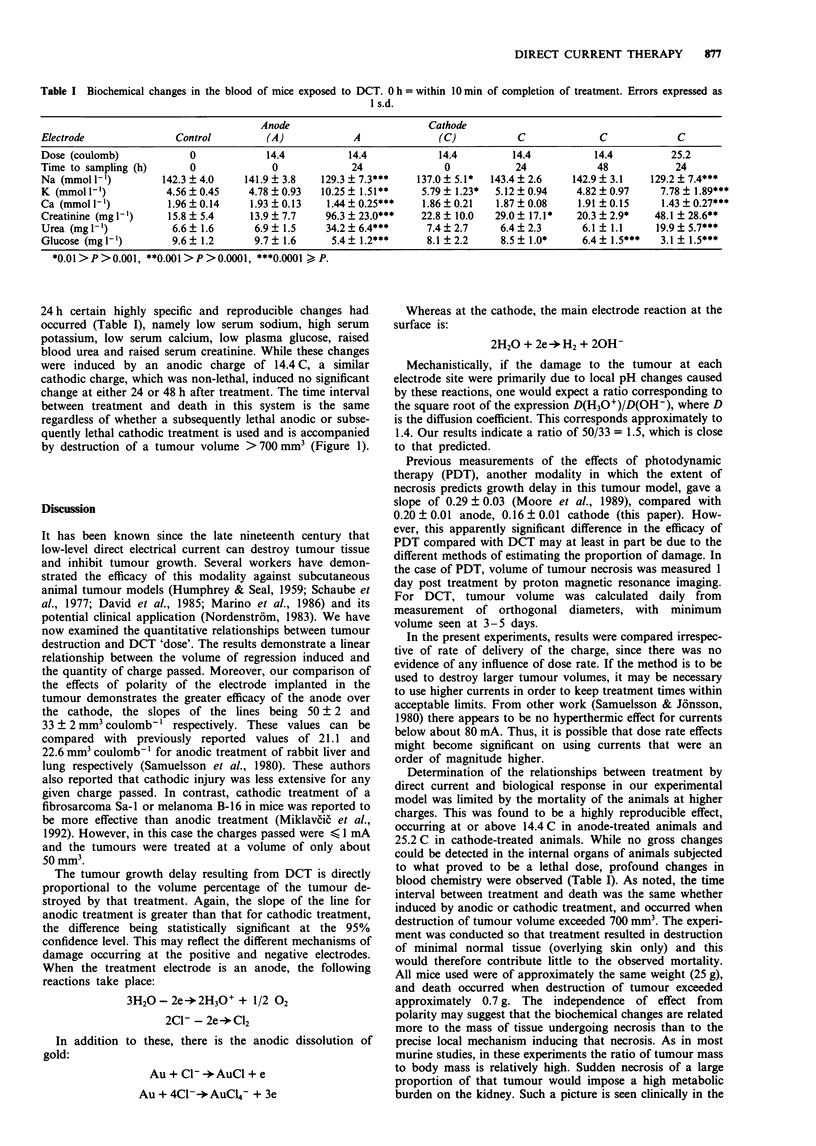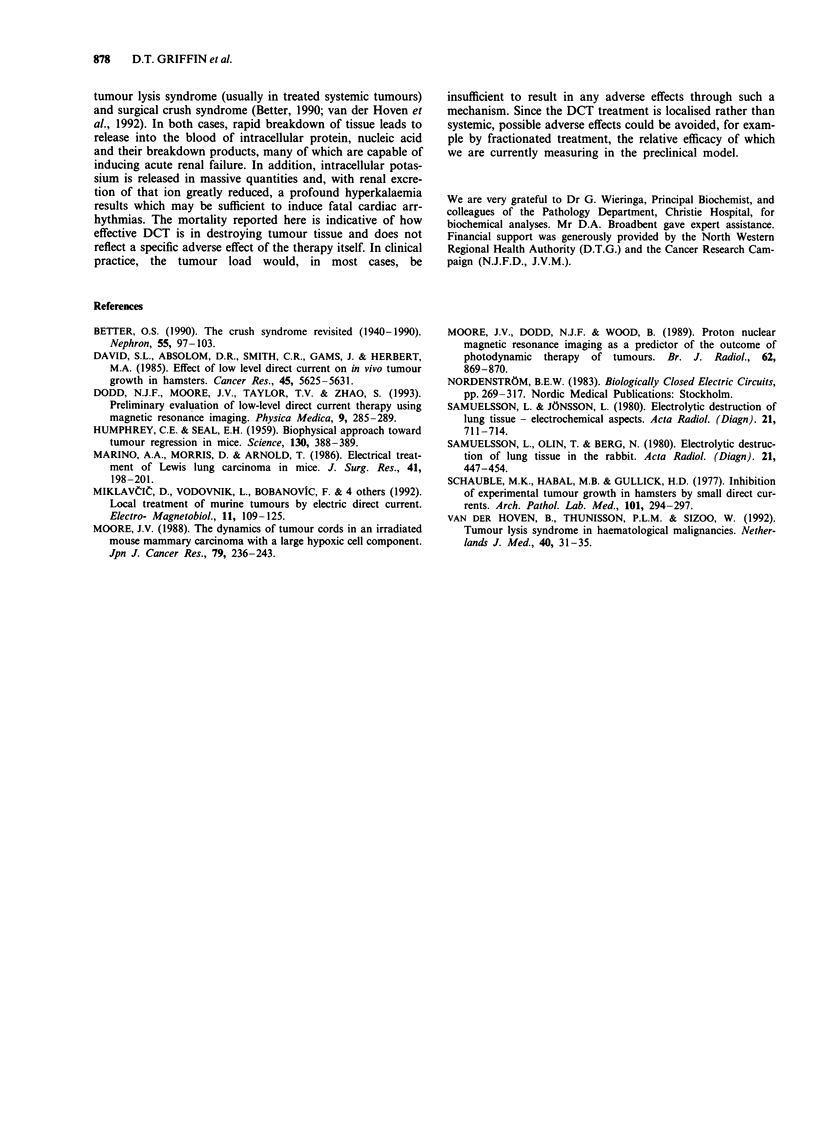# The effects of low-level direct current therapy on a preclinical mammary carcinoma: tumour regression and systemic biochemical sequelae.

**DOI:** 10.1038/bjc.1994.169

**Published:** 1994-05

**Authors:** D. T. Griffin, N. J. Dodd, J. V. Moore, B. R. Pullan, T. V. Taylor

**Affiliations:** Paterson Institute for Cancer Research, Christie Hospital (NHS) Trust, Manchester, UK.

## Abstract

Low-level direct electric current has been shown to be capable of destroying tumour tissue. Using an early-passage subcutaneous murine mammary carcinoma, the relationships between the volume of tumour destruction, charge and polarity have been examined. The results revealed a direct correlation between charge passed and absolute volume regression when the intratumoral electrode was made either an anode or a cathode. Tumour destruction for a given charge was significantly greater following anodic than cathodic treatment. A direct correlation was also observed between the percentage volume of prompt treatment-induced regression and the in situ end point of tumour growth delay. During the course of these experiments, a highly reproducible toxic effect was discovered, which has not been previously reported for this modality. An anodic charge greater than 10.6 coulombs or a cathodic charge greater than 21.6 coulombs resulted in 100% mortality at 24-72 h, while lower charges had no influence on mortality. Quantitative assays of a number of blood parameters showed that mortality was associated with serum electrolyte imbalances and appeared to be the result of the metabolic load of tumour breakdown products. These effects are similar to the tumour lysis or surgical crush syndromes and should not constitute a significant problem in clinical practice, where the tumour mass to total body mass ratio will normally be much smaller.


Br. J. Cancer (1994), 69, 875-878 © Macmillan Press Ltd., 1994

### The effects of low-level direct current therapy on a preclinical mammary carcinoma: tumour regression and systemic biochemical sequelae

D.T. Griffin[1], N.J.F. Dodd[1], J.V. Moore[1], B.R. Pullan[2] & T.V. Taylor[2]

[1]*Paterson Institute for Cancer Research, Christie Hospital (NHS) Trust, Manchester M20 9BX, UK;* [2]*Department of Surgical Gastroenterology, Manchester Royal Infirmary, Manchester M13 9WL, UK.*

**Summary** Low-level direct electric current has been shown to be capable of destroying tumour tissue. Using an early-passage subcutaneous murine mammary carcinoma, the relationships between the volume of tumour destruction, charge and polarity have been examined. The results revealed a direct correlation between charge passed and absolute volume regression when the intratumoral electrode was made either an anode or a cathode. Tumour destruction for a given charge was significantly greater following anodic than cathodic treatment. A direct correlation was also observed between the percentage volume of prompt treatment-induced regression and the *in situ* end point of tumour growth delay. During the course of these experiments, a highly reproducible toxic effect was discovered, which has not been previously reported for this modality. An anodic charge greater than 10.6 coulombs or a cathodic charge greater than 21.6 coulombs resulted in 100% mortality at 24-72 h, while lower charges had no influence on mortality. Quantitative assays of a number of blood parameters showed that mortality was associated with serum electrolyte imbalances and appeared to be the result of the metabolic load of tumour breakdown products. These effects are similar to the tumour lysis or surgical crush syndromes and should not constitute a significant problem in clinical practice, where the tumour mass to total body mass ratio will normally be much smaller.

Direct current therapy (DCT) offers considerable promise as a low-cost, minimally invasive anti-tumour treatment. While the tissue-destructive effects of low, direct electrical currents have been known for many years, development of a clinically acceptable therapy has been slow, hindered, for example, by uncertainties regarding the quantitation of the dose-response relationship. Our previous qualitative study demonstrated that both anodic and cathodic treatments caused prompt and massive tumour necrosis (Dodd *et al.*, 1993). The present work provides an absolute and relative quantitation of the extent of tumour regression/necrosis with charge and polarity. In common with previous workers, we noted that tumour lysis and volume decrease was extremely rapid after DCT. For other therapies, it has been observed clinically that an undesirable 'tumour lysis syndrome' may result in these circumstances (van der Hoven *et al.*, 1992). Accordingly, we also examine here the potential systemic consequences of treating tumours by DCT.

### Materials and methods

All procedures to be described complied with the Animals (Scientific Procedures) Act 1986 (UK). Male B6DF2$_1$ (Paterson) mice, 6-8 weeks old, weight approximately 25 g, were inoculated subcutaneously with a suspension of cells from a low-passage, syngeneic murine mammary carcinoma, T50/80 (Moore, 1988). Tumours were treated by DCT 6-10 weeks after inoculation when they were approximately spherical and 6-10 mm in diameter. Tumour size was assessed using vernier calipers to measure three orthogonal diameters $a$, $b$ and $c$. Tumour volume was calculated using the approximation:

$$V \approx \pi abc/6$$

Up to four mice were treated simultaneously while held under general anaesthesia and maintained at a constant temperature of 37°C. Anaesthesia was induced by i.p. injection of ketalar (Park Davis, Pontypool, UK) and maintained using halothane (ICI Pharmaceuticals, Alderley Edge, UK) and oxygen inhalation for the duration of the procedure. Each animal was placed on a copper plate electrode covered in conducting gel (Dracard, Maidstone, UK). The second electrode consisted of a 1 cm length of gold wire, 0.25 mm diameter, spot welded to an 8 cm length of phosphor-bronze wire. This was inserted into the tumour through a 21 G needle which was subsequently removed, and held firmly by a supporting gantry above the mouse with the terminal gold section *only* in contact with the tumour. A new gold electrode was used after every 2-3 treatments, thus minimising the effects of gradual roughening of the electrode surface due to dissolution of gold when the electrode was made an anode. Before use as anode or cathode, these intratumoral electrodes were sterilised with 70% alcohol. Direct current was passed between the electrodes by means of a computer-controlled, constant-current power supply which continually monitored voltage. The power source used here was designed to maintain a constant current; applied voltage was therefore automatically varied accordingly within the range 1-16 V. For an implanted anode, current and duration of application varied from 1 to 4 mA and 30 to 90 min respectively. For the cathode, values were 1-5 mA and 30-90 min. Different limits of total charge delivered during anodic and cathodic treatments were imposed by mortality of the animals (see below). Animals were randomly allocated to a current-time-polarity treatment group, with seven test animals and one control animal per group. Controls were exposed to anaesthesia and implantation of the electrode, but no current was passed. After treatment animals were housed in individual cages. Each tumour was measured daily until it had grown to 125% of its pretreatment volume, when the animal was killed using a schedule 1 method.

The initial volume of control tumours varied over the same range as did treated tumours, and this enabled data from controls to be used in a computer program that generated idealised tumour growth curves for any initial tumour volume within that range. Using this program, (i) maximum decrease in tumour volume and (ii) tumour regrowth delay were calculated for each treated tumour, relative to the size-matched control curve. In some cases the decrease in volume was equal to the total initial volume, and in a few cases no regrowth of the tumour was observed, even after several months, i.e. the animals were 'cured' of their tumours. However, in this first series, only those tumours in which partial damage (i.e. less than total volume regression) occurred were used to quantify the effects of treatment. In those tumours in which the relative volume regression was 100%, the *absolute* volume regression attainable for that dose was unknown.

Correspondence: J.V. Moore.
Received 13 September 1993; and in revised form 12 January 1994.

Using the same methods as above, further groups of animals were treated with higher charges. Groups were either (i) sacrificed at different times after treatment and their organs subjected to histological examination or (ii) placed under deep terminal anaesthesia, blood taken and biochemical analysis of blood parameters carried out ('Cobras Mira-S' analyser; Roche, Welwyn Garden City, UK).

Statistical methods used were as follows: For calculation of volume regression versus charge and growth delay versus percentage volume regression, a linear–linear regression yielded the best (least-squares) fit to the data. Analysis of biochemical data (treated–untreated) was made by a two-sample $t$-test, significance being taken as $P = 95\%$ or greater.

### Results

### *Anodic treatment*

When volume regression was analysed against various parameters of dose delivered, the best correlation was obtained when plotted against *charge*, although it did appear that higher currents resulted in greater volume regression for the same charge passed, possibly because of higher rates of production of electrolytic products (see Discussion). Regression analysis of the data showed a linear relationship between the volume of regression induced in the tumour and the charge passed when the electrode in the tumour was an anode (Figure 1). The line of best fit is given by the linear equation:

$$V = (50 \pm 2)C + (18 \pm 15) \qquad (r = 0.930,\ P_{\text{slope}} < 0.001,\ n = 76)$$

Where $V$ is the volume in $\text{mm}^3$ and $C$ is the charge in coulombs. A measure of the efficacy of an anti-tumour therapy in experimental animals is provided by the growth delay induced in the tumour by that therapy. With a localised therapy such as DCT one would expect growth delay to be dependent on the proportion of the tumour volume destroyed. Regression analysis of the experimental data showed a linear relationship, the line of best fit being given by the equation:

$$G = (0.20 \pm 0.01)D + 1.4 \pm 0.5 \qquad (r = 0.925,\ P_{\text{slope}} < 0.001,\ n = 76)$$

where $G$ is the growth delay in days and $D$ is the maximum percentage volume decrease.

### *Cathodic treatment*

Regression analysis of the data obtained on cathodic treatment of the tumours again showed a linear relationship (Figure 1) between volume of tumour regression and charge. The line of best fit is given by the equation:

$$V = (33 \pm 2)C + 38 \pm 16 \qquad (r = 0.87,\ P_{\text{slope}} < 0.001,\ n = 108)$$

Similarly, for growth delay and percentage volume decrease:

$$G = (0.16 \pm 0.01)D + 2.1 \pm 0.4 \qquad (r = 0.85,\ P_{\text{slope}} < 0.001,\ n = 108)$$

Note that when the gold electrode was made a cathode, the remote copper plate, which then became an anode, showed evidence of dissolution with (non-traumatic) traces of a green cupric chloride deposit visible on the animal's skin by the end of treatment.

### *Treatment-induced mortality*

During the course of the experiments described above, the effects of higher dose could not be examined, owing to mortality of the animals. Consequently, experiments were carried out to determine the onset and cause of this mortality which, to our knowledge, has not been previously reported. Tumour-bearing animals were randomly assigned to one of eight groups, each containing six mice. Two groups were used as controls and underwent anaesthesia and implantation

**Figure 1** Volume regression (pretreatment minus nadir) induced in T50/80 tumours as a function of electrical charge passed, with the inserted electrode either anodic (**a**) or cathodic (**b**). Individual data points and linear least-squares fit.

of the electrode, but no current was passed. The remaining groups received either anodic or cathodic treatment with 4 mA for 60 min (14.4 C), 3 mA for 90 min (16.2 C) or 7 mA for 60 min (25.2 C). All animals made a good recovery from the anaesthetic and treated animals could not be distinguished from controls for the first 12 h. From 12 h onwards, all anode-treated groups and the 25.2 C cathode-treated group began to exhibit signs of abnormal behaviour, becoming hypothermic with decreased motor tone. From incidental deaths in the earlier experiments, it was known that all these animals would die within 72 h of treatment, with most animals dying between 24 and 48 h. Animals in the other groups showed no untoward effects. Histological examination of internal organs was performed on further groups of animals, treated in the same way but sacrificed within 10 min of completion of treatment (0 h) or at 12, 24, 48 or 72 h later. However, no macroscopic or light microscopic signs of organ damage could be found to explain the observed toxic effects.

### *Biochemical analysis of blood*

Animals were randomly allocated to one of seven groups, each containing 16 mice. The treatments received by each group are shown in Table I. At specified times after treatment, blood was taken under terminal anaesthesia and analysed biochemically for a number of clinically relevant parameters (Table I). Immediately after treatment with a subsequently lethal dose via either anode or cathode, the blood profile varied little from that of control animals. By

**Table I** Biochemical changes in the blood of mice exposed to DCT. 0 h = within 10 min of completion of treatment. Errors expressed as 1 s.d.

| *Electrode* | *Control* | *Anode (A)* | *A* | *Cathode (C)* | *C* | *C* | *C* |
|---|---|---|---|---|---|---|---|
| Dose (coulomb) | 0 | 14.4 | 14.4 | 14.4 | 14.4 | 14.4 | 25.2 |
| Time to sampling (h) | 0 | 0 | 24 | 0 | 24 | 48 | 24 |
| Na (mmol $l^{-1}$) | 142.3 ± 4.0 | 141.9 ± 3.8 | 129.3 ± 7.3*** | 137.0 ± 5.1* | 143.4 ± 2.6 | 142.9 ± 3.1 | 129.2 ± 7.4*** |
| K (mmol $l^{-1}$) | 4.56 ± 0.45 | 4.78 ± 0.93 | 10.25 ± 1.51** | 5.79 ± 1.23* | 5.12 ± 0.94 | 4.82 ± 0.97 | 7.78 ± 1.89*** |
| Ca (mmol $l^{-1}$) | 1.96 ± 0.14 | 1.93 ± 0.13 | 1.44 ± 0.25*** | 1.86 ± 0.21 | 1.87 ± 0.08 | 1.91 ± 0.15 | 1.43 ± 0.27*** |
| Creatinine (mg $l^{-1}$) | 15.8 ± 5.4 | 13.9 ± 7.7 | 96.3 ± 23.0*** | 22.8 ± 10.0 | 29.0 ± 17.1* | 20.3 ± 2.9* | 48.1 ± 28.6** |
| Urea (mg $l^{-1}$) | 6.6 ± 1.6 | 6.9 ± 1.5 | 34.2 ± 6.4*** | 7.4 ± 2.7 | 6.4 ± 2.3 | 6.1 ± 1.1 | 19.9 ± 5.7*** |
| Glucose (mg $l^{-1}$) | 9.6 ± 1.2 | 9.7 ± 1.6 | 5.4 ± 1.2*** | 8.1 ± 2.2 | 8.5 ± 1.0* | 6.4 ± 1.5*** | 3.1 ± 1.5*** |

*$0.01 > P > 0.001$, **$0.001 > P > 0.0001$, ***$0.0001 \geqslant P$.

24 h certain highly specific and reproducible changes had occurred (Table I), namely low serum sodium, high serum potassium, low serum calcium, low plasma glucose, raised blood urea and raised serum creatinine. While these changes were induced by an anodic charge of 14.4 C, a similar cathodic charge, which was non-lethal, induced no significant change at either 24 or 48 h after treatment. The time interval between treatment and death in this system is the same regardless of whether a subsequently lethal anodic or subsequently lethal cathodic treatment is used and is accompanied by destruction of a tumour volume $> 700 \text{ mm}^3$ (Figure 1).

### Discussion

It has been known since the late nineteenth century that low-level direct electrical current can destroy tumour tissue and inhibit tumour growth. Several workers have demonstrated the efficacy of this modality against subcutaneous animal tumour models (Humphrey & Seal, 1959; Schaube *et al.*, 1977; David *et al.*, 1985; Marino *et al.*, 1986) and its potential clinical application (Nordenström, 1983). We have now examined the quantitative relationships between tumour destruction and DCT 'dose'. The results demonstrate a linear relationship between the volume of regression induced and the quantity of charge passed. Moreover, our comparison of the effects of polarity of the electrode implanted in the tumour demonstrates the greater efficacy of the anode over the cathode, the slopes of the lines being $50 \pm 2$ and $33 \pm 2 \text{ mm}^3 \text{ coulomb}^{-1}$ respectively. These values can be compared with previously reported values of 21.1 and $22.6 \text{ mm}^3 \text{ coulomb}^{-1}$ for anodic treatment of rabbit liver and lung respectively (Samuelsson *et al.*, 1980). These authors also reported that cathodic injury was less extensive for any given charge passed. In contrast, cathodic treatment of a fibrosarcoma Sa-1 or melanoma B-16 in mice was reported to be more effective than anodic treatment (Miklavčič *et al.*, 1992). However, in this case the charges passed were $\leqslant 1$ mA and the tumours were treated at a volume of only about $50 \text{ mm}^3$.

The tumour growth delay resulting from DCT is directly proportional to the volume percentage of the tumour destroyed by that treatment. Again, the slope of the line for anodic treatment is greater than that for cathodic treatment, the difference being statistically significant at the 95% confidence level. This may reflect the different mechanisms of damage occurring at the positive and negative electrodes. When the treatment electrode is an anode, the following reactions take place:

$$3H_2O - 2e \rightarrow 2H_3O^+ + 1/2\ O_2$$

$$2Cl^- - 2e \rightarrow Cl_2$$

In addition to these, there is the anodic dissolution of gold:

$$Au + Cl^- \rightarrow AuCl + e$$

$$Au + 4Cl^- \rightarrow AuCl_4^- + 3e$$

Whereas at the cathode, the main electrode reaction at the surface is:

$$2H_2O + 2e \rightarrow H_2 + 2OH^-$$

Mechanistically, if the damage to the tumour at each electrode site were primarily due to local pH changes caused by these reactions, one would expect a ratio corresponding to the square root of the expression $D(H_3O^+)/D(OH^-)$, where $D$ is the diffusion coefficient. This corresponds approximately to 1.4. Our results indicate a ratio of $50/33 = 1.5$, which is close to that predicted.

Previous measurements of the effects of photodynamic therapy (PDT), another modality in which the extent of necrosis predicts growth delay in this tumour model, gave a slope of $0.29 \pm 0.03$ (Moore *et al.*, 1989), compared with $0.20 \pm 0.01$ anode, $0.16 \pm 0.01$ cathode (this paper). However, this apparently significant difference in the efficacy of PDT compared with DCT may at least in part be due to the different methods of estimating the proportion of damage. In the case of PDT, volume of tumour necrosis was measured 1 day post treatment by proton magnetic resonance imaging. For DCT, tumour volume was calculated daily from measurement of orthogonal diameters, with minimum volume seen at 3–5 days.

In the present experiments, results were compared irrespective of rate of delivery of the charge, since there was no evidence of any influence of dose rate. If the method is to be used to destroy larger tumour volumes, it may be necessary to use higher currents in order to keep treatment times within acceptable limits. From other work (Samuelsson & Jönsson, 1980) there appears to be no hyperthermic effect for currents below about 80 mA. Thus, it is possible that dose rate effects might become significant on using currents that were an order of magnitude higher.

Determination of the relationships between treatment by direct current and biological response in our experimental model was limited by the mortality of the animals at higher charges. This was found to be a highly reproducible effect, occurring at or above 14.4 C in anode-treated animals and 25.2 C in cathode-treated animals. While no gross changes could be detected in the internal organs of animals subjected to what proved to be a lethal dose, profound changes in blood chemistry were observed (Table I). As noted, the time interval between treatment and death was the same whether induced by anodic or cathodic treatment, and occurred when destruction of tumour volume exceeded $700 \text{ mm}^3$. The experiment was conducted so that treatment resulted in destruction of minimal normal tissue (overlying skin only) and this would therefore contribute little to the observed mortality. All mice used were of approximately the same weight (25 g), and death occurred when destruction of tumour exceeded approximately 0.7 g. The independence of effect from polarity may suggest that the biochemical changes are related more to the mass of tissue undergoing necrosis than to the precise local mechanism inducing that necrosis. As in most murine studies, in these experiments the ratio of tumour mass to body mass is relatively high. Sudden necrosis of a large proportion of that tumour would impose a high metabolic burden on the kidney. Such a picture is seen clinically in the tumour lysis syndrome (usually in treated systemic tumours) and surgical crush syndrome (Better, 1990; van der Hoven *et al.*, 1992). In both cases, rapid breakdown of tissue leads to release into the blood of intracellular protein, nucleic acid and their breakdown products, many of which are capable of inducing acute renal failure. In addition, intracellular potassium is released in massive quantities and, with renal excretion of that ion greatly reduced, a profound hyperkalaemia results which may be sufficient to induce fatal cardiac arrhythmias. The mortality reported here is indicative of how effective DCT is in destroying tumour tissue and does not reflect a specific adverse effect of the therapy itself. In clinical practice, the tumour load would, in most cases, be insufficient to result in any adverse effects through such a mechanism. Since the DCT treatment is localised rather than systemic, possible adverse effects could be avoided, for example by fractionated treatment, the relative efficacy of which we are currently measuring in the preclinical model.

We are very grateful to Dr G. Wieringa, Principal Biochemist, and colleagues of the Pathology Department, Christie Hospital, for biochemical analyses. Mr D.A. Broadbent gave expert assistance. Financial support was generously provided by the North Western Regional Health Authority (D.T.G.) and the Cancer Research Campaign (N.J.F.D., J.V.M.).

### References

BETTER, O.S. (1990). The crush syndrome revisited (1940–1990). *Nephron*, **55**, 97–103.

DAVID, S.L., ABSOLOM, D.R., SMITH, C.R., GAMS, J. & HERBERT, M.A. (1985). Effect of low level direct current on *in vivo* tumour growth in hamsters. *Cancer Res.*, **45**, 5625–5631.

DODD, N.J.F., MOORE, J.V., TAYLOR, T.V. & ZHAO, S. (1993). Preliminary evaluation of low-level direct current therapy using magnetic resonance imaging. *Physica Medica*, **9**, 285–289.

HUMPHREY, C.E. & SEAL, E.H. (1959). Biophysical approach toward tumour regression in mice. *Science*, **130**, 388–389.

MARINO, A.A., MORRIS, D. & ARNOLD, T. (1986). Electrical treatment of Lewis lung carcinoma in mice. *J. Surg. Res.*, **41**, 198–201.

MIKLAVČIČ, D., VODOVNIK, L., BOBANOVÍC, F. & 4 others (1992). Local treatment of murine tumours by electric direct current. *Electro- Magnetobiol.*, **11**, 109–125.

MOORE, J.V. (1988). The dynamics of tumour cords in an irradiated mouse mammary carcinoma with a large hypoxic cell component. *Jpn J. Cancer Res.*, **79**, 236–243.

MOORE, J.V., DODD, N.J.F. & WOOD, B. (1989). Proton nuclear magnetic resonance imaging as a predictor of the outcome of photodynamic therapy of tumours. *Br. J. Radiol.*, **62**, 869–870.

NORDENSTRÖM, B.E.W. (1983). *Biologically Closed Electric Circuits*, pp. 269–317. Nordic Medical Publications: Stockholm.

SAMUELSSON, L. & JÖNSSON, L. (1980). Electrolytic destruction of lung tissue – electrochemical aspects. *Acta Radiol. (Diagn)*. **21**, 711–714.

SAMUELSSON, L., OLIN, T. & BERG, N. (1980). Electrolytic destruction of lung tissue in the rabbit. *Acta Radiol. (Diagn)*. **21**, 447–454.

SCHAUBLE, M.K., HABAL, M.B. & GULLICK, H.D. (1977). Inhibition of experimental tumour growth in hamsters by small direct currents. *Arch. Pathol. Lab. Med.*, **101**, 294–297.

VAN DER HOVEN, B., THUNISSON, P.L.M. & SIZOO, W. (1992). Tumour lysis syndrome in haematological malignancies. *Netherlands J. Med.*, **40**, 31–35.